# Autonomic dysreflexia associated with cervical spinal cord gliofibroma: case report

**DOI:** 10.1186/s12883-021-02271-z

**Published:** 2021-06-29

**Authors:** Hiroyuki Mizuno, Fumiaki Honda, Hayato Ikota, Yuhei Yoshimoto

**Affiliations:** 1 Departments of Neurosurgery, Gunma University Graduate School of Medicine, 3-39-22 Showa-machi, Gunma 371-8511 Maebashi, Japan; 2 Departments of Human Pathology, Gunma University Graduate School of Medicine, Gunma Maebashi, Japan

**Keywords:** Autonomic dysreflexia, Gliofibroma, Cervical spinal cord tumor

## Abstract

**Background:**

Autonomic dysreflexia (AD) is an abnormal reflex of the autonomic nervous system normally observed in patients with spinal cord injury from the sixth thoracic vertebra and above. AD causes various symptoms including paroxysmal hypertension due to stimulus. Here, we report a case of recurrent AD associated with cervical spinal cord tumor.

**Case presentation:**

The patient was a 57-year-old man. Magnetic resonance imaging revealed an intramedullary lesion in the C2, C6, and high Th12 levels. During the course of treatment, sudden loss of consciousness occurred together with abnormal paroxysmal hypertension, marked facial sweating, left upward conjugate gaze deviation, ankylosis of both upper and lower extremities, and mydriasis. Seizures repeatedly occurred, with symptoms disappearing after approximately 30 min. AD associated with cervical spinal cord tumor was diagnosed. Histological examination by tumor biopsy confirmed the diagnosis of gliofibroma. Radiotherapy was performed targeting the entire brain and spinal cord. The patient died approximately 3 months after treatment was started.

**Conclusions:**

AD is rarely associated with spinal cord tumor, and this is the first case associated with cervical spinal cord gliofibroma. AD is important to recognize, since immediate and appropriate response is required.

## Background

Autonomic dysreflexia (AD) is an abnormal reflex of the autonomic nervous system that is primarily observed in patients with spinal cord injury located at the sixth thoracic vertebra and above [1]. AD is well known to cause various symptoms including paroxysmal hypertension due to stimulus [2, 3]. Here, we report a rare case of recurrent AD associated with a cervical spinal cord tumor.

## Case presentation

### History and examination

**Fig. 1:**
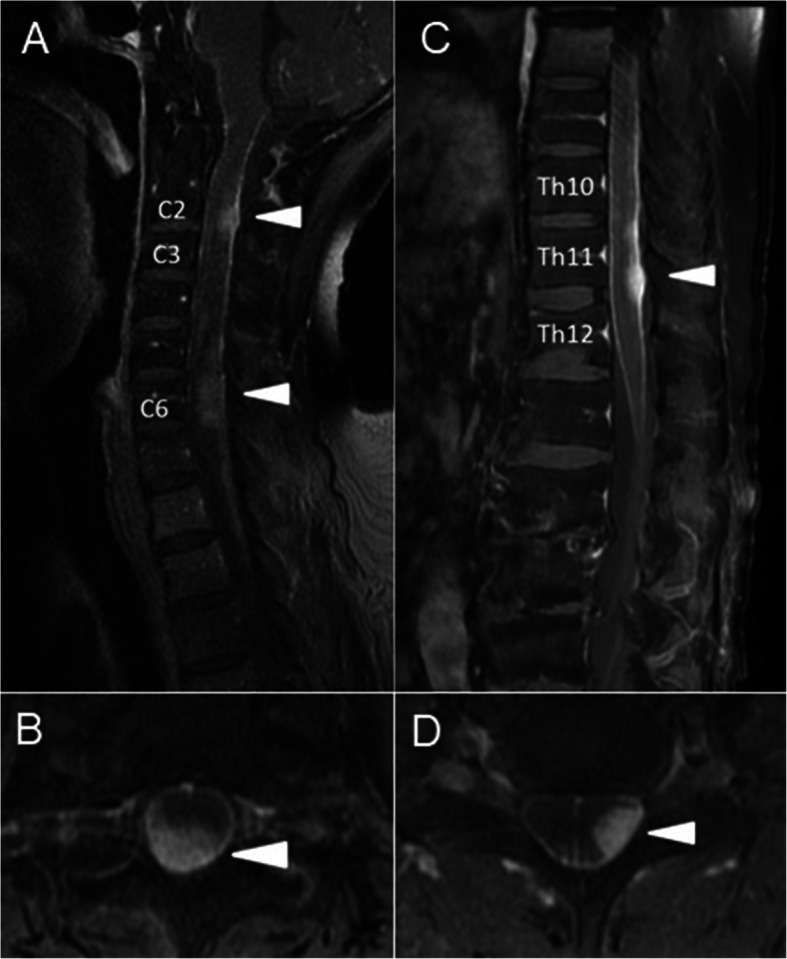
T1-weighted magnetic resonance image with gadolinium on admission showing multiple contrast-enhanced lesions in the posterior intramedullary spinal cord at the C2 and C6 levels and on the spinal cord surface at the C7 and C8 levels (**A**, **B**), and in the left posterior intramedullary spinal cord at the Th12 level and on the surface of the spinal cone (**C**, **D**)

A 57-year-old man presented with right lower extremity pain and light headedness when standing up. He had no related family history or medical history. His right leg pain gradually worsened and movement became difficult over a period of 4 months, so he was transferred by ambulance. On admission, he was aware and conscious, and complained of nervous pain in the right lower leg. Physical examination found mild paralysis of manual muscle test 4/5 and increased deep tendon reflex in the left half of the body. Orthostatic hypotension was observed in which systolic blood pressure decreased to approximately 70 mmHg when sitting. In addition, mild urination and defecation disorders were noted. Cerebrospinal fluid examination indicated elevated initial pressure of 27 cmH_2_O and high protein level of 605 mg/dl. No tumor cells were detected in the cerebrospinal fluid, and collagen-related antibody levels were normal. Magnetic resonance imaging revealed contrast-enhanced lesions at the posterior intramedullary C2 and high C6 levels, left posterior intramedullary Th12 level, medullary cone surface, and left middle cerebellar peduncle (Fig. 1).

### Postadmission course

On the day after admission, the patient suddenly lost consciousness and systolic blood pressure rose to around 230 mmHg, with marked facial sweating, upper conjugate deviation of the left eye, ankylosis of both upper and lower limbs, and dilated pupils. His head was immediately elevated and head computed tomography/magnetic resonance imaging was performed but found no abnormalities. Intravenous injection of nicardipine hydrochloride was performed to reduce his blood pressure, but his blood pressure was refractory and difficult to reduce immediately. This neurological symptom could not be explained by a transient ischemic attack, so antithrombotic therapy was not started. Epileptic seizures were suspected, and diazepam (10 mg) and fosphenytoin sodium hydrate (750 mg) were administered intravenously. Conjugate deviation of the eye and ankylosis in both upper and lower extremities were alleviated, but the hypertension, facial sweating, and dilated pupils showed almost no improvement. However, these symptoms gradually disappeared after approximately 30 min to one hour, and the patient’s consciousness disturbance was ameliorated. Such attacks repeatedly occurred 1 to 5 times daily and were mainly triggered by pain during physical activity and daily nursing care. Electroencephalography in the resting state recorded on day 2 showed α waves predominantly originating from the occipital lobe at 8–10 Hz, but no laterality was observed. Complex partial epileptic seizures were suspected, so oral administration of lacosamide (100 mg/day) was initiated on the same day for prophylaxis. However, the frequency of seizures remained unchanged.

Sudden onset of symptoms of sympathetic hyperactivity accompanied by consciousness disorder triggered by pain indicated AD associated with an intramedullary tumor. Initially, the number of therapeutic agents was increased and more care was taken to minimize the stimulation caused when changing postures and moving the bed, which acted as triggers. However, non-steroidal anti-inflammatory drugs had little analgesic effect on the resting right lower extremity pain, so oral administration of carbamazepine 400 mg/day was initiated, together with administration of tofisopam (150 mg/day) and gabapentin (1200 mg/day). The patient had presented with bladder-rectal disorder at the time of admission, so catheterization of the urethra was performed and oral administration of laxatives and prokinetic agents for constipation was initiated. However, the seizures repeatedly occurred, so early treatment was planned. Tumor biopsy was performed at the Th10-11 level on hospital day 3.

## Operative findings

**Fig. 2:**
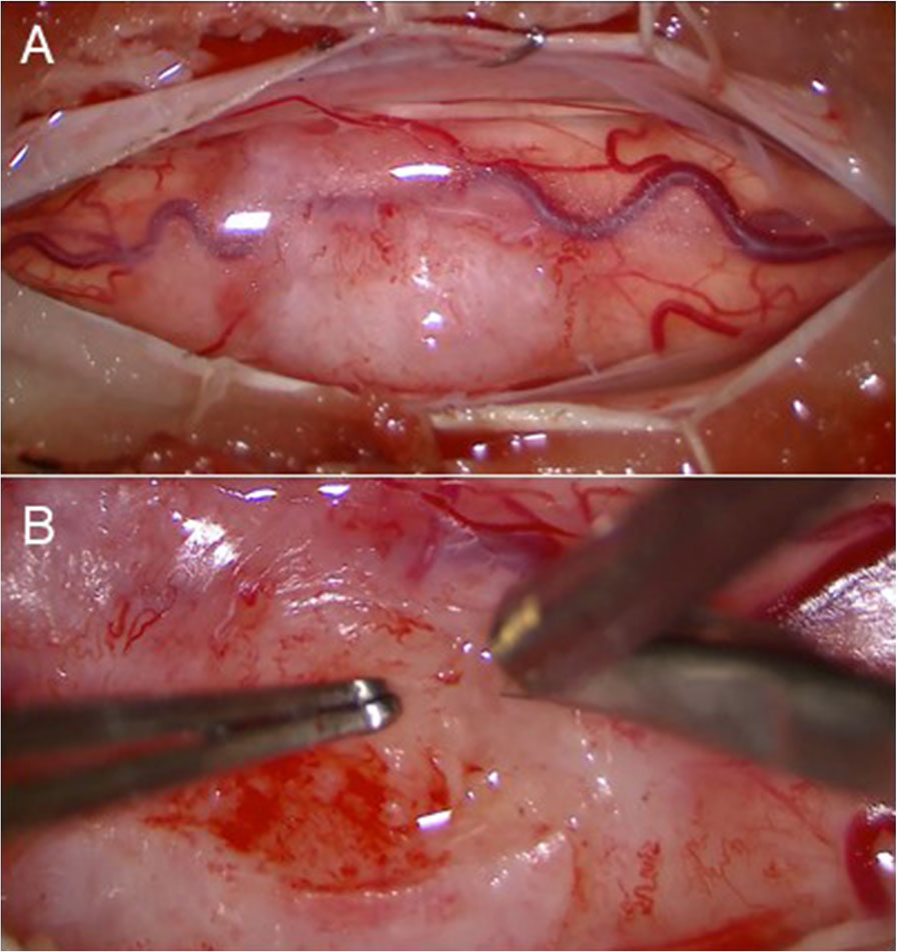
Intraoperative photographs showing the tumor was elastic hard with a capsule, white surface, and no adhesion to the dura, and extended exophytically from the left side of the dorsal column of the spinal cord (Upper). The tumor was relatively hypovascular and the boundary between the tumor tissue and normal spinal cord was not clear (Lower)

No adhesion of the tumor to the dura was observed. The tumor was white on the surface, elastic hard with a capsule, and hypovascular. The capsule structure disappeared in the deep part of the tumor, and the boundary between the tumor and the normal spinal cord was unclear. Therefore, the tumor appeared to be intramedullary (Fig. 2). The operation was completed after only collecting tissue for diagnosis.

### Histopathological findings

**Fig. 3:**
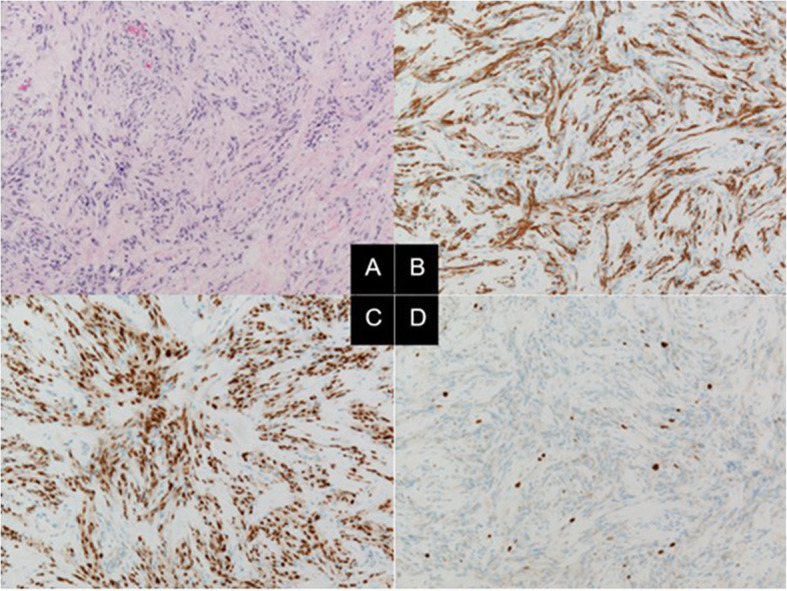
Pathological findings. **A**: Hematoxylin and eosin staining showing tumor cells with short spindle-shaped nucleus and eosinophilic cytoplasm proliferated in bundles and intricate shapes with collagenous interstitium. Original magnification x100. **B** and **C**: Immunohistochemical staining showing positive results for glial fibrillary acidic protein in the cytoplasm (**B**) and for Olig2 in the nucleus (**C**). **D**: MIB-1 staining showing labeling index of about 2 %

Tumor cells with short spindle-shaped nuclei and eosinophilic cytoplasm proliferated in bundles, forming an intricate pattern along with collagenous fibrous interstitial tissue. No increase in mitotic figures, microvessel proliferation, or necrosis was observed. Immunohistochemical examination showed the tumor cells were S-100 protein (+), glial fibrillary acidic protein (+), Olig2 (+), mIDH1-R132H (-), p53 (+, less than 10 %), and ATRX (+), with MIB-1 labeling index of 2 % (Fig. 3).

### Postoperative course

**Fig. 4:**
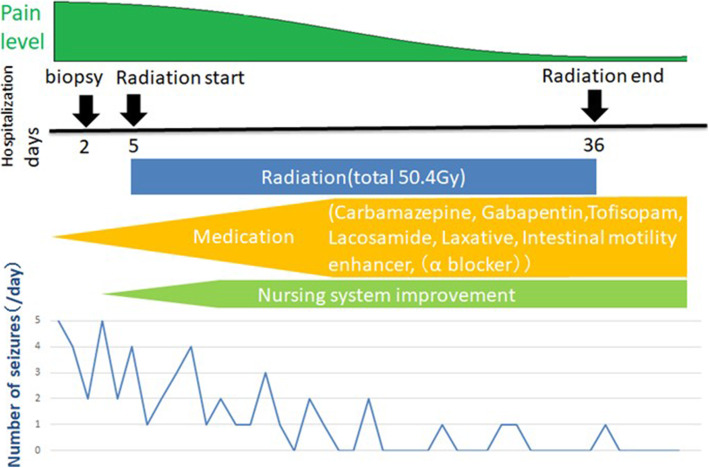
Treatment course, pain level, and number of seizures. The pain and seizure frequency gradually decreased with drug treatment, improvement of the nursing system, and start of radiation therapy, but the seizures could not be completely suppressed

The frequency of AD was gradually reduced by adjusting the nursing system, alleviating pain, and initiating oral pharmacotherapy (Fig. 4). The histological diagnosis was gliofibroma, with few findings suggesting malignancy. However, imaging revealed disseminated lesions spreading from the entire spinal cord to the posterior fossa. Irradiation 36 Gy was administered to the whole spinal cord and posterior fossa, and 12.6 Gy to the local lesion in the cerebellum. However, the tumors showed little change, and the disseminated lesions gradually spread. The patient died approximately 3 months after the course of treatment was completed.

## Discussion and conclusion

### Autonomic dysreflexia

AD is a paroxysmal abnormal reflex of the autonomic nerves occurring in patients with spinal cord injury of Th6 and above, initially reported in 1917 [4]. AD has been defined as uncontrollable transient increase in blood pressure of 20 mmHg or more [5, 6]. The clinical manifestations are diverse, and include abnormal autonomic hyperactivity symptoms such as sudden hypertension, dilated pupils, headache, and bradycardia, in addition to sweating, piloerection, rubefaction, nasal congestion of non-paralyzed skin, chest pain, nausea, and vomiting [2, 7]. The compensatory mechanism fails with severe seizures and hyperperfusion occurs, causing consciousness disorder/convulsive seizures, occasional hypertensive encephalopathy, and cerebral hemorrhage [8]. Other serious life-threatening complications such as fatal arrhythmia require urgent diagnosis and treatment [9, 10]. Complications during the chronic stage may lead to deterioration of cardiac function, cognitive impairment, and other pulmonary, retinal, and renal disorders, so the prognosis is poor [11–13].

Onset is generally reported to occur most frequently during the chronic phase after spinal cord injury (3–6 months after injury) [3], with up to 40 seizures daily [14]. AD rarely occurs in patients with spinal cord tumor and non-traumatic spinal cord disease such as multiple sclerosis [15, 16]. Only one case of AD has been reported in cervical intramedullary astrocytoma with onset associated with spinal cord tumor [15]. The most common seizure-inducing factor is bladder/rectal tension due to bladder-rectal dysfunction associated with autonomic neuropathy, but various other stimuli may also be inducing factors [2, 17, 18].

### Mechanism

**Fig. 5:**
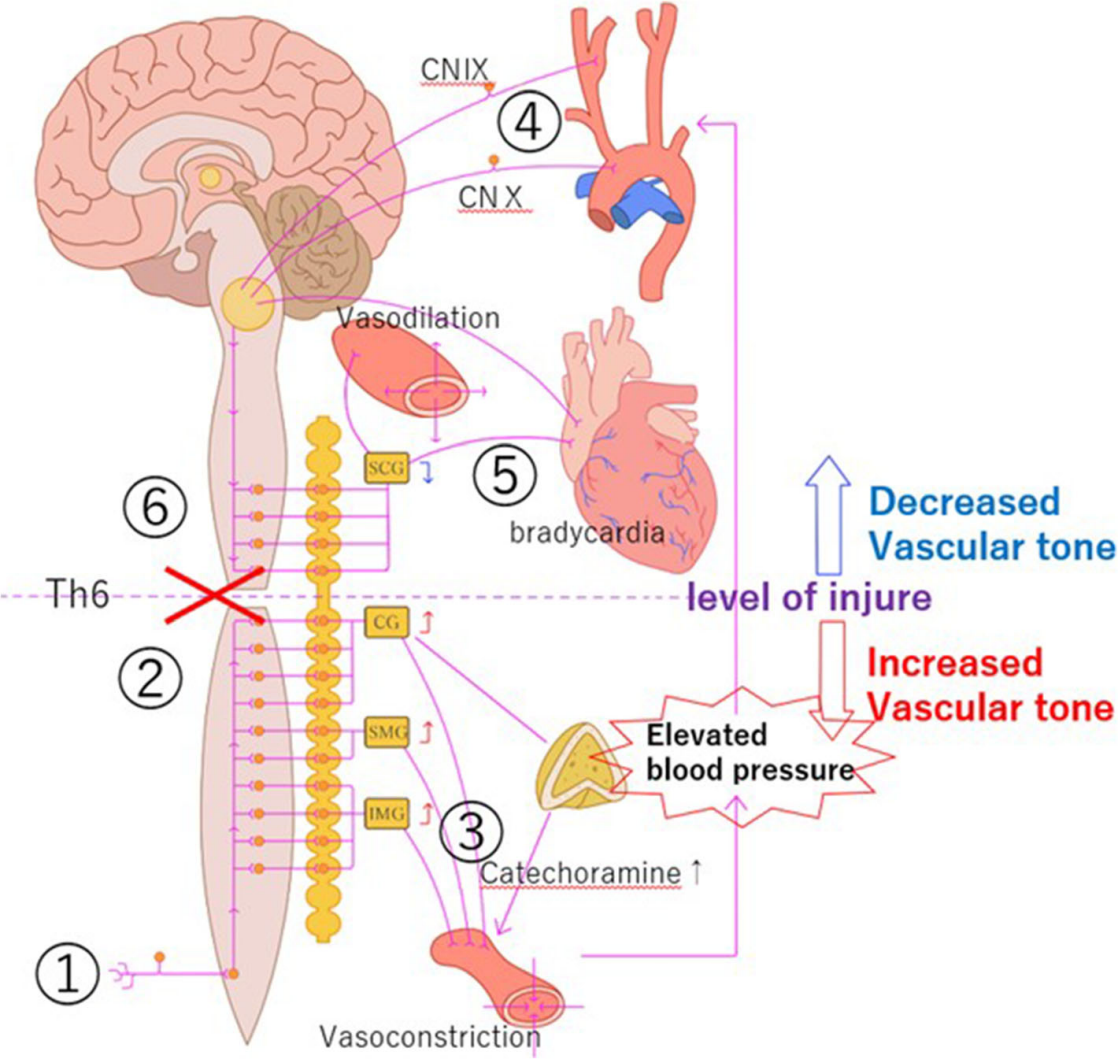
Seizure mechanism. Vasoconstriction below the injury level and vasodilation above the injury level was caused by t created using Adobe Illustrator. he spinal cord injury above the Th6 cord. This vascular tone gap caused abnormal hypertension, dilated pupils, headaches, sweating, and flushing. This figure was created using Adobe Illustrator

The mechanism for onset is as follows [19]. The trigger stimulus passes up the spinal cord via the higher sensory branches to reach the brain, and is also transmitted to the sympathetic nerve system (greater splanchnic nerve) branching from the Th4-L2 levels, resulting in sympathetic nerve-mediated vasoconstriction and increased blood pressure. This systemic increase in blood pressure caused by the pain trigger stimulates the baroreceptors of the carotid sinus and aortic arch, resulting in vagal bradycardia and central vasodilation of the vasomotor nerves. Such increase in blood pressure is immediately controlled by this series of compensatory mechanisms in healthy individuals. However, the vagal descending pathway is disrupted in patients with spinal cord injury, so that no inhibitory signal is transmitted below the level of the spinal cord injury and consequently the vasoconstriction continues. Blood vessels above the level of spinal cord injury undergo vasodilation as a strong inhibitory signal is transmitted. Therefore, vasoconstriction below the injury level and vasodilation above the injury level occur simultaneously, and the vascular tone gap results in abnormal hypertension, dilated pupils, headache, sweating, and flushing. This abnormal reflex arc is not resolved and hypertension persists unless the stimulus ceases (Fig. 5). At the same time, the sympathetic ganglia also act on the adrenal cortex, resulting in raised serum adrenaline and noradrenaline concentrations, and promotion of hypertension [20]. The α1 and α2 receptors are up-regulated in the peripheral blood vessels of AD patients and vascular hypersensitivity to catecholamines is increased, and this higher hypersensitivity contributes to the severity of the hypertension [21].

### Treatment

#### Elimination of the cause and prophylaxis

AD-induced hypertension is an abnormal reflex arc which is not relieved unless the causative stimulus is eliminated. Therefore, identification of the cause is the most important treatment step [22]. However, the seizure-inducing factors differ from case to case, so individual analysis of the causes is important to treat individual patients. Bladder/rectal tightness due to bladder-rectal injury is the most common symptom, so urinary retention should be confirmed, or catheter obstruction should be excluded if an indwelling urethral catheter has been inserted [18]. Oral administration of anticholinergic drugs and gabapentin is effective for preventing intestinal tract/bladder tension [23, 24], and botox injection into the bladder/rectal sphincter and surgical treatment were successful in severe cases [25–28]. However, the onset of AD cannot be completely inhibited only by elimination and prevention of the causes, so oral pharmacotherapy and preparation for response to seizures are necessary.

#### Oral pharmacotherapy

Nifedipine, a typical Ca blocker, is the most commonly used oral drug to relieve seizures [12, 29, 30], but requires care in long-term administration because AD patients are hypotensive due to autonomic neuropathy [31, 32]. Prazosin, a selective alpha-blocker, does not exert a rapid inhibitory effect on cardiac function and resting blood pressure, so can significantly reduce the severity of AD-related seizures [33, 34]. In addition, oral administration of nitroglycerin, hydralazine, captopril, prostaglandin E2, terazosin, clonidine, and hydralazine reduce the frequency and severity of seizures [29, 35–37]. The sympatholytic drugs, guanethidine and tofisopam, normally used for autonomic neuropathy, and gabapentin, sometimes used for neuropathic pain, are also reported to reduce the frequency of seizures [38].

#### Emergency response

Emergent reduction of cerebral perfusion pressure can be achieved by raising the head of the patient [34]. Antihypertensive agents are administered as needed [18, 39]. Nitroglycerin, hydralazine, captopril, and other agents have been recommended as antihypertensive agents in emergencies [10, 30, 40]. Frequent blood pressure monitoring (every 2–5 min) is important because blood pressure may change rapidly [41].

#### Gliofibroma

Gliofibroma is a rare histological type defined in the 4th edition of the Classification of Tumours of the Central Nervous System of the World Health Organization in 2007. Gliofibroma consists of both glial and mesenchymal components. The age of onset is 11 to 54 years (median: 14 years), and the male-female ratio is 2:3. The most common sites are the cerebrum (36 %) and spinal cord (28 %). The glioma component may be low or high grade, but the mesenchymal component has no malignant characteristics. The histological types that should be excluded during differential diagnosis include desmoplastic infantile astrocytoma and ganglioglioma, pleomorphic xanthoastrocytoma, and gliosarcoma. Gliofibroma has a good prognosis, but dissemination and death have been reported in cases with anaplastic glioma components and increased mitotic Figs. [42, 43].

#### Present case

The present patient had gliofibroma located in high spinal positions, at the C2, C6, and Th12 levels. Severe AD repeatedly occurred due to neural pain in the right buttock. Radiotherapy was initiated immediately after biopsy was performed. Since the primary trigger for AD was neuropathic pain, gabapentin, which is administered for neuropathic pain and may have protective effects against AD, was orally administered in addition to carbamazepine, resulting in gradual alleviation of the pain. In addition, we reviewed the nursing system and minimized the irritation associated with movement. Oral administration of laxatives and prokinetic agents reduced bladder/intestinal irritation. In addition, internal administration of tofisopam and lacosamide was started. This combined treatment achieved gradual decrease in seizure frequency, but not complete inhibition. Administration of an alpha-blocker might also be appropriate to consider.

## Conclusions

The present case of gliofibroma manifested with extremely rare sympathetic hypertonic reflex associated with cervical spinal cord tumor. AD is rarely associated with spinal cord tumor, and this is the first case associated with cervical spinal cord gliofibroma. AD is important to recognize, since immediate and appropriate response is required.

## Data Availability

Not applicable.

## Abbreviations

AD: Autonomic dysreflexia
ATRX: α-Thalassemia/mental retardation syndrome X-linked
MIB-1: Mind bomb homolog-1
mIDH1: Mutations in isocitrate dehydrogenase 1
Olig2: Oligodendrocyte lineage transcription factor 2
